# Prise en charge des orbitopathies dysthyroidiennes modérées et sévères: à propos de 22 cas

**DOI:** 10.11604/pamj.2017.27.257.13008

**Published:** 2017-08-07

**Authors:** Nadia Daldoul, Leila Knani, Faten Gatfaoui, Hechmi Mahjoub

**Affiliations:** 1 Service d'Ophtalmologie, CHU Farhat Hached, Sousse, Tunisie

**Keywords:** Ophtalmopathie basedowienne, neuropathie, exophtalmie, décompression, corticoïdes, Basedow-graves ophthalmopathy, neuropathy, exophthalmos decompression, corticosteroids

## Abstract

Décrire la prise en charge thérapeutique des orbitopathies dysthyroidiennes modérées et sévères et évaluer par une étude statistique les facteurs associés à la neuropathie optique ainsi que les facteurs de mauvais pronostic visuel. Nous avons mené une étude rétrospective sur 22 patients présentant une ophtalmopathie dysthyroidienne modérée à sévère sur au moins un oeil, hospitalisés au service d'ophtalmologie du CHU Farhat Hached Sousse, sur une période s'étalant de 1998 à 2015. Les indications thérapeutiques sont basées sur les critères d'activité et de sévérité de l'Eugogo ainsi que l'évaluation des facteurs de mauvais pronostic visuel. L'âge moyen de nos patients était de 40 ans avec une légère prédominance masculine (54.5%). 68.2% des patients étaient en euthyroidie, 18.2% étaient tabagique. Le facteur le plus associé significativement à la neuropathie est la compression au niveau de l'apex orbitaire (P = 0.03). Le traitement était basé sur la corticothérapie intraveineuse et/ou la décompression orbitaire en fonction de l'activité et la sévérité de la maladie. L'évolution globale après traitement a été marquée par une amélioration des signes inflammatoires, réduction de l'exophtalmie. Le pronostic visuel était plus mauvais chez les patients plus âgés (P = 0.0001), de sexe masculin (P = 0.03) et traités par irathérapie (P = 0.04). Dans les limites d'une étude rétrospective, nos résultats étaient globalement concordants avec la littérature. L'orbitopathie dysthyroidienne reste une maladie dont l'évaluation et la prise en charge thérapeutique sont non encore bien élucidées. Des études de cohortes, probablement multicentriques, sont à envisager pour améliorer la prise en charge.

## Abstract (en)

Our study aimed to describe the therapeutic management of moderate and severe dysthyroid orbitopathies and to evaluate the factors associated with optic neuropathy as well as the prognostic factors of poor visual outcome using a statistical analysis. We conducted a retrospective study of 22 patients presenting with moderate to severe dysthyroid orbitopathy in at least one eye and hospitalized in the Department of Ophthalmology at the University Hospital Farhat Hached, Sousse over the period from 1998 to 2015. Therapeutic indications were based on activity and severity criteria of the Eugogo (European Group On Graves' Orbitopathy) as well as on the assessment of prognostic factors of poor visual outcome. The average age of our patients was 40 years, with a slight male predominance (54.5%). 68.2% of patients were euthyroid, 18.2% had a history of smoking. The most significantly associated factor with neuropathy was a compression in the orbital apex (p = 0.03). Treatment was based on intravenous corticosteroid therapy and/or orbital decompression based on disease activity and severity. Overall evolution after treatment was marked by an improvement in inflammatory signs and by the reduction of exophthalmia. Visual prognosis was worse in elderly patients (p = 0.0001), male sex (p = 0.03) and treated by iratherapy (p = 0.04). Given the limits of a retrospective study, our results were generally consistent with the literature. The assessment and the management of dysthyroid orbitopathy are not well understood. Cohort, probably multicenter studies should be conducted to improve its management.

## Introduction

L'orbitopathie dysthyroidienne est une maladie auto-immune qui représente la manifestation extra-thyroïdienne la plus fréquente. Sa physiopathologie est imparfaitement élucidée, elle est le résultat d'une auto-réactivité croisée entre antigènes thyroïdien et tissu orbitaire. Elle peut se voir dans un contexte d'hyperthyroïdie le plus fréquemment (maladie de basedow ou graves disease) parfois d'hypothyroïdie (thyroïdite d'hashimoto) ou d'euthyroidie [1]. Généralement les formes minimes ont un retentissement fonctionnel minime et sont de résolution spontanée alors que les formes modérées 5-10% des cas entrainent une gène fonctionnelle et une altération de la qualité de vie des patients et les formes sévères (3-5 % des cas) menacent le pronostic visuel à très court terme par neuropathie optique ou atteinte cornéenne [1]. Pour ces formes sévères et jusqu'à ce jour, il n'y a pas de consensus thérapeutique. S'il y a unanimité sur les immunosuppresseurs et les corticoïdes, plusieurs protocoles thérapeutiques sont disponibles. Nous avons mené une étude rétrospective sur 22 patients présentant une ophtalmopathie dysthyroidienne modérée et sévère, hospitalisés au service d'ophtalmologie du CHU Farhat Hached Sousse sur une période s'étalant entre 1998 et 2015. Les objectifs de ce travail étaient de: décrire la prise en charge thérapeutique et les résultats des orbitopathies dysthyroidiennes modérées et sévères suivies au service d'ophtalmologie de Sousse; évaluer par une étude statistique les facteurs associés à la neuropathie optique et les facteurs de mauvais pronostic visuel.

## Méthodes

Il s'agit d'une étude rétrospective portant sur 22 patients présentant une ophtalmopathie dysthyroidienne modérée et sévère. Nous avons inclus tous les patients ayant une ophtalmopathie dysthyroidienne, dont au moins un œil présente une ophtalmopathie modérée ou sévère selon le score de sévérité de la classification de l'EUGOGO (European Group On Grave's Orbitopathy) [2]. Le diagnostic d'orbitopathie dysthyroidienne a été retenu sur des critères de l'association américaine de la thyroïde (ATA) [3]. Nous avons exclu les patients qui présentaient une inflammation orbitaire d'autre origine (infectieuse, tumorale, vasculaire ou inflammation orbitaire idiopathique). Les données étaient recueillies à partir des dossiers médicaux des patients. Un interrogatoire minutieux précisant en particulier: l'âge, le sexe, habitude de vie: tabac, type de trouble thyroïdien, traitement reçu (Iode radioactif, chirurgie thyroïdienne, traitement anti thyroïdien, traitement hormonal substitutif), motif de consultation. L'examen ophtalmologique a permis de quantifier le score d'activité et de sévérité de l'ophtalmopathie dysthyroidienne selon le score établi par l'EUGOGO. Les examens complémentaires incluent une TDM cérébro-orbitaire qui permet de quantifier l'exophtalmie, rechercher une hypertrophie musculaire ou graisseuse, une IRM cérébro-orbitaire permettant l'étude du signal musculaire et la recherche d'une compresssion au niveau de l'apex orbitaire. En cas de suspicion de neuropathie optique: un relevé du champ visuel a été demandé (Périmétrie Cinétique de Goldmann) Les tests biologiques comportaient un dosage de TSH, T3, T4, Anticorps anti R-TSH. Nous avons retenu le diagnostic de neuropathie optique devant la présence des critères suivants: baisse visuelle, atteinte du reflexe photomoteur afférent, œdème ou pâleur papillaire au fond dœil(FO), altération périmétrique ou signes de compression au niveau de l'apex. La prise en charge du dysfonctionnement thyroïdien était réalisée en collaboration avec les endocrinologues.

Le volet ophtalmologique reposait sur des mesures générales: sevrage tabagique, une administration de larmes artificielles, antibiotiques fortifiés en cas d'abcès cornéen, l'administration de corticoides et si besoin immunosuppresseur (cyclosporine 3-5 mg/kg) en cas de corticorésistance. Deux protocoles de glucocorticoides ont été utilisés: soit administration de méthylprednisolone IV à la dose de 1g pendant 3 jours consécutifs puis relais aux corticoïdes par voie orale (1mg/kg) et dégression progressive sur 2 mois soit le deuxiéme protocole: 500 mg de méthylprednisolone administrée par voie intraveineuse sur 3 jours consécutifs, sur 4 cycles de 4 semaines d'intervalle (la dose cumulative était de 6g). Le traitement chirurgical nécessite une euthyroidie clinique et biologique de 6 mois ainsi qu'une orbitopathie dysthyroidienne stabilisée et inactive, sauf pour les formes menaçant le pronostic visuel (neuropathie compressive corticorésistante). La décompression orbitaire peut intéresser différents parois osseuses (le plancher orbitaire de part et d'autre du nerf infraorbitaire et/ou la paroi médiale et/ou la paroi latérale et/ou la graisse orbitaire en utilisant une incision cutanée sous ciliaire à 3mm du bord libre palpébral inférieur. Pour les rétractions palpébrales modérées, nous avons injecté la toxine botulique en deux points d'injection, par voie conjonctivale, à la dose de 5 UI à 10 UI au niveau de chaque paupière supérieure. L’allongement du muscle releveur de la paupière supérieure a été réalisé par greffe d’aponévrose temporale. Dans un cas de rétraction palpébrale majeure, une blépharotomie de pleine épaisseur a été réalisée chez un patient. Pour l'allongement de la paupière inférieure, nous avons eu recours soit à un allongement par lambeau nasogénien ou par récession des rétracteurs et greffe dermique. L'évaluation du pronostic visuel chez tous les patients a été basée sur deux critères suivants (une acuité visuelle ≥ 6/10 avec papille normale au fond d’œil (pour les deux yeux) après traitement). Il a été jugé bon si ces deux critères étaient présents.

**Saisie et analyse des données**: La saisie et l'analyse des données ont été réalisées avec le logiciel SPSS version 17. Les tests statistiques utilisés étaient: le test « t » de Student pour échantillons appariés pour la comparaison des moyennes en prenant comme seuil de signification la valeur de 5%.

## Résultats

L'âge moyen de notre population était de 40 ans avec une légère prédominance masculine: 12 hommes (54.5%) contre 10 femmes (45.5%). Seulement, quatre patients étaient fumeurs (18.2%). La majorité des patients (19 patients sur 22) avait des antécédents de maladie de Basedow (86.4%). Trois patients (13.6%) avaient une ophtalmopathie dysthyroidienne sans maladie thyroïdienne connue antérieure (les anticorps anti récepteurs à la TSH ont été demandés et sont revenus négatifs). Lors de l'hospitalisation, 15 patients étaient traités par des antithyroïdiens de synthèse (68.2%), 8 patients (36.4%) avaient reçu une IRAthérapie (thérapie à l'iode radioactif), 9 patients (40.9%) étaient sous traitement substitutif et 4 patients (18.2%) avaient subi une thyroïdectomie.

**Facteurs associés à la neuropathie**: 50% des patients avec SAC >3 avaient une neuropathie optique (P = 1). L'imagerie orbitaire montrait une compression au niveau de l'apex dans 85.71 % des yeux (P = 0.03), une hypertrophie graisseuse et musculaire à des proportions égales de 44% des cas (P = 0.89) et un étirement du nerf optique dans 11.7% des yeux (P = 1).

**Prise en charge thérapeutique**: La prise en charge de la dysthyroidie était réalisée par les endocrinologues. Les doses thérapeutiques ont été adaptées chez 5 patients (22.7%). La prise en charge ophtalmologique était différente selon le score de sévérité et d'activité. Notre série comprenait 10 patients avec orbitopathie modérée et 12 patients avec orbitopathie sévère (Tableau 1).

**Tableau 1:** : Etude clinique et classification selon la sévérité et l’activité

Classification	Active	Inactive	Total
Orbitopathie dysthyroidienne modérée	3	7	10
Orbitopathie dysthyroidienne sévère	4	8	12
Total	7	15	22

### Orbitopathie dysthyroidienne modérée

**Figure 1:**
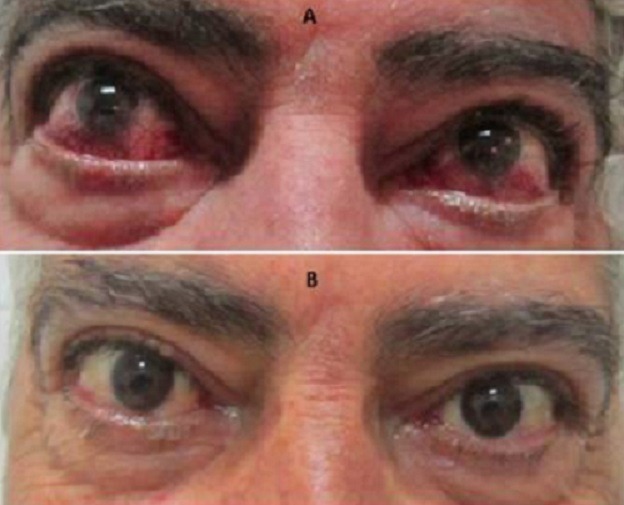
Orbitopathie dysthyroidienne avec hyperhémie conjonctivale ODG: (A) avant corticothérapie; (B) aspect après traitement

**Orbitopathie dysthyroidienne modérée active (3 patients)**: Le traitement de l'OD modérée active était basé sur l'administration de corticoïdes par voie intraveineuse (3 patients). Parmi ces patients, un patient présentait une hypertonie oculaire résistante à la quadrithérapie, avec excavation papillaire rapidement progressive. On a constaté une réduction du score d'activité clinique qui a passé de 3 à 1 chez 2 patients, une réduction de l'exophtalmie ainsi qu'une amélioration de l'oculomotricité (Figure 1). Chez le troisième patient, une décompression orbitaire (à type de décompression inféromédiale) a été nécessaire à cause de l'hypertonie oculaire qui n'a pas répondu à la quadrithérapie et aux corticoïdes (1g). L'évolution post opératoire a été favorable en association à un traitement hypotonisant. Nous avons renoncé à la chirurgie filtrante après la décompression osseuse (normalisation des chiffres tensionnels).

**Figure 2:**
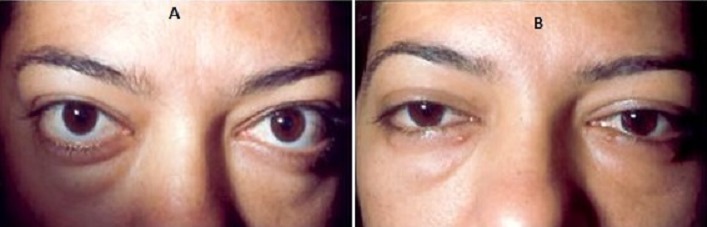
Décompression à but esthétique (exophtalmie modérée): aspect satisfaisant après 10 mois: (A) aspect avant chirurgie; (B) aspect post opératoire

**Figure 3:**
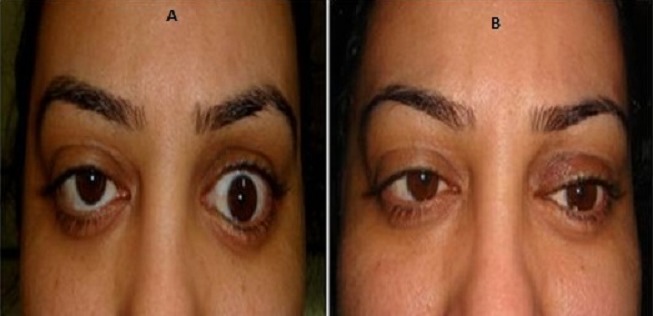
Rétraction de la paupière supérieure gauche: (A) avant traitement; (B) après injection de toxine botulique

**Figure 4:**
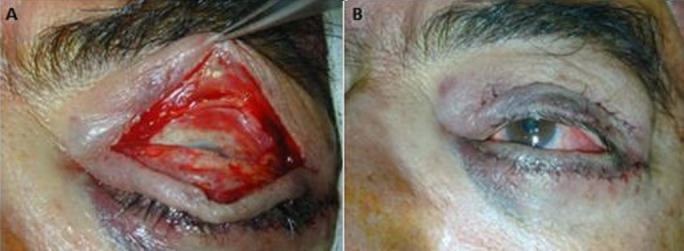
Blépharotomie pour rétraction palpébrale supérieure: (A) aspect per opératoire; (B) résultat satisfaisant après intervention

**Figure 5:**
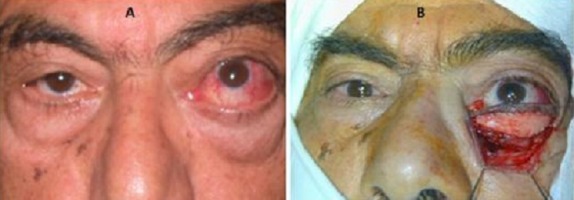
Greffe dermique pour rétraction palpébrale inférieure: (A) aspect préopératoire; (B) aspect per opératoire de greffe dermique

**Orbitopathie dysthyroidienne modérée inactive (7 patients)**: La décompression orbitaire a été indiquée chez 6 patients (11 yeux): à but esthétique pour 4 patients présentant une exophtalmie isolée et à but fonctionnel pour 2 patients (exophtalmie+diplopie chez un patient, exophtalmie + kératite superficielle pour l'autre). Nous avons utilisé une décompression osseuse pour 11 yeux, associée à une résection de la graisse extraconique pour 4 yeux. La décompression osseuse incluait le plancher de l'orbite (2 yeux), la paroi latérale et médiale (2 yeux), la paroi inféromédiale (5 yeux) ou les 3 parois de l'orbite (2 yeux). Réalisée à but esthétique (4 patients), le résultat post opératoire était bon avec diminution de l'exophtalmie et bonne occlusion chez 3 patients (Figure 2). Une persistance d'une rétraction palpébrale supérieure avec apparition d'une complication post opératoire transitoire à type de diplopie a été notée chez le quatrième. Pour les deux patients qui ont été opérés pour une gêne fonctionnelle, on avait une diminution de la diplopie chez un patient et une occlusion incomplète pour les deux yeux chez l'autre patient, nécessitant le recours à un allongement palpébral ultérieur. Nous avons eu recours à l'injection de toxine botulique chez 2 patients (4 yeux). Une injection était indiquée pour rétraction palpébrale modérée avec amélioration de la rétraction mais cette amélioration était transitoire (Figure 3). Une autre patiente a été injectée pour rétraction persistante après décompression orbitaire. La toxine botulique n'a pas montré d'effet ce qui a nécessité une chirurgie palpébrale ultérieure. L'allongement palpébral a été pratiqué chez 3 patients (4 yeux). L'indication de la chirurgie palpébrale était une persistance de lagophtalmie après chirurgie orbitaire. La technique utilisée consistait en une blépharotomie de la paupière supérieure (1 oeil) (Figure 4), un allongement palpébral par greffe dermique (2 yeux) (Figure 5) et un allongement par aponévrose temporale (2 yeux). Pour les patients (2 yeux) ayant eu greffe dermique: un allongement par aponévrose temporale suturée au tarse et à l'aponévrose du releveur de paupière supérieure (1 œil) ) ainsi qu’une reprise par utilisation d'un lambeau nasogénien (1 œil) étaient nécessaire suite à une nécrose ultérieure du greffon. L'évolution finale de cette chirurgie palpébrale a été marquée par une bonne occlusion palpébrale finale chez tous les yeux.

### Orbitopathie dysthyroidienne sévère (12 patients)

**Neuropathie optique (9 patients):** Nous avons utilisé les corticoïdes par voie veineuse comme traitement de première intention chez les sujets avec neuropathie optique. Le recours à la cyclosporine après non réponse aux corticoïdes a été réalisé chez 2 patients pour persistance d’œdème papillaire et apparition de paleur papillaire respectivement. Une décompression orbitaire a été indiquée chez 4 patients pour persistance d'un œdème papillaire après corticothérapie (1oeil), début d'installation de pâleur papillaire avec persistance d'altérations périmétriques (5 yeux). La décompression osseuse (6 yeux) a été associée à une résection graisseuse dans tous les yeux. La technique utilisée pour la décompression consistait en un effondrement des 3 parois (2 yeux), un effondrement de la paroi inféro médiale (4 yeux) L'acuité visuelle finale dans notre étude était ≥ 6/10 dans 41.2% des yeux. Pour 4 yeux, nous avons noté une disparition complète des anomalies périmétriques. Le fond d'oeil a révélé la persistance d'une pâleur papillaire dans 2 yeux. La cyclosporine a donné un bon résultat chez le patient présentant un œdème papillaire avec résolution des signes d'activité, amélioration de l'acuité visuelle et résorption de l’œdème papillaire. L'association corticoïdes-cyclosporine chez le deuxième patient a entrainé une diminution de l'exophtalmie avec résolution des signes inflammatoires mais elle s'est montrée inefficace sur la neuropathie optique avec persistance d'une pâleur papillaire incitant une décompression urgente. On a constaté après décompression osseuse une amélioration de la vision chez 2 patients avec un retour à un aspect normal de la papille et normalisation des altérations périmétriques. 2 patients (2 yeux) n'ont pas été améliorés après décompression avec persistance de pâleur papillaire post opératoire. On a constaté une seule complication à type d'hypoesthésie maxillaire en réalisant une décompression inféromédiale.

**Figure 6:**
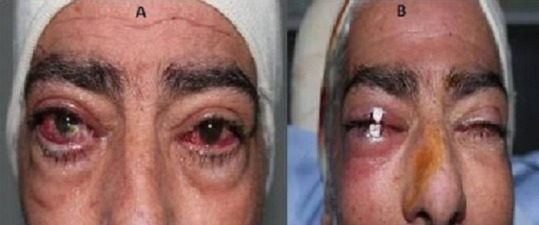
Prise en charge d'abcès cornéen: (A) abcès cornéen droit; (B) blépharorraphie transitoire pour lagophtalmie

**Atteinte cornéenne (3 patients)**: Les patients qui avaient une atteinte à type d'abcès cornéens (11.4%) ont été traités par larmes artificielles et blépharorraphie (Figure 6). Ils ont reçu des antibiotiques par voie locale en fonction du germe identifié. L'évolution a été marquée par un nettoyage de l'abcès chez tous les patients avec persistance d'ulcère trophique chez un patient qui avait une cornée décompensée et une hypotropie résiduelle qui favorisait l'exposition cornéenne.

**Pronostic visuel**: On a retrouvé que 11 patients (50%) avaient un bon pronostic visuel. Le pronostic visuel était plus mauvais chez les patients plus âgés (P = 0.0001), de sexe masculin (P = 0.03) et traités par irathérapie (P = 0.04).

## Discussion

**Neuropathie optique et facteurs associés**: La survenue d'une neuropathie optique dysthyroidienne (NO) est rare 5% [2]. Elle met en jeu le pronostic visuel et nécessite une prise en charge urgente et adaptée. L'évaluation et la prise en charge de NO dysthyroidienne constitue un sujet à controverses ce qui a amené le comité d'EUGOGO à standardiser l'évaluation clinique en performant des études multicentriques [3]. En effet, le diagnostic de NO n'est pas toujours aisé et il n'existe pas de critères bien définis pour la neuropathie optique dysthyroidienne et qu'elle nécessite un faisceau d'arguments [4–6] (acuité visuelle, œdème ou paleur du disc optique, altération de vision des couleurs et du champs visuel, compression au niveau de l'apex). Les études multicentriques réalisées par l'EUGOGO [3] retrouvaient que la moyenne de score d'activité clinique (SAC) est de 4/7, soit 25% ont un score < 3, indiquant que l'inflammation sévère n'est pas obligatoire pour entrainer une NO. Dans notre étude, on a trouvé que 38.09% des patients ayant une NO avaient un SAC < 3. Dans notre série, 50% des patients avec SAC>3 ont une neuropathie optique mais il n'existe pas de corrélation significative entre score d'activité clinique et neuropathie optique (P = 1). On n'a pas montré de relation significative entre l'hypertrophie graisseuse et la neuropathie optique (P = 0.89). M.Al Bakri [7] partage la même constatation, il concluait que l'hypertrophie graisseuse entraine un étirement du nerf optique, le volume du nerf optique reste le même mais il devient moins incurvé. Cet étirement du nerf optique n'a pas de rôle dans le développement de neuropathie optique. De même, bien que plusieurs études suggèrent que l'élargissement musculaire est un important critère dans le diagnostic de neuropathie optique [8], des cas de neuropathie optique sans hypertrophie musculaire ont été rapportés [9]. Le mécanisme le plus communément admis de la neuropathie optique est l'encombrement apical par élargissement des muscles oculomoteurs [7, 10]. On a retrouvé une corrélation très significative entre l'existence de signes de compression du nerf optique en imagerie et l'existence de neuropathie optique clinique (P=0.03). Mais, ce score n'est pas à lui seul le facteur responsable de développement de neuropathie optique.

**Prise en charge thérapeutique**: Les protocoles thérapeutiques de l'orbitopathie dysthyroidienne (OD) sont très variés et dépendent essentiellement du score de sévérité et du caractère actif ou non de l'orbitopathie.

### Orbitopathie dysthyroidienne modérées

**Orbitopathie dysthyroidienne modérée active**: Les corticoïdes systémiques à forte dose constituent le traitement de première ligne des OD modérée actives [11]. Les protocoles sont très diverses et il n'existe pas de consensus international. Le régime le plus commun de traitement IV de glucocorticoïdes en se basant sur une étude randomisée [12, 13] correspond à une dose cumulative de 4.5 g de méthylprednisolone répartie sur 12 semaines. Dans notre série, on a constaté une réduction du score d'activité clinique qui a passé de 3 à 1 chez 2 patients, une réduction de l'exophtalmie ainsi qu'une amélioration de l'oculomotricité. En fait, l'effet des corticoïdes a été évalué par des études randomisées [14] qui ont constaté une amélioration des symptômes dans 69% des cas, inactivation de score d'activité clinique dans 59-89% des cas et une amélioration de l'oculomotricité dans 57% des cas.

### Orbitopathie dysthyroidienne modérée inactive

**Décompression orbitaire**: Les incisions chirurgicales dans la décompression orbitaire sont variées: coronale, au niveau du plis palpébral supérieur, au niveau du canthus latéral, au niveau du fornix inférieur, tanscaronculaire [15]. On a utilisé dans notre série l'incision transpalpébrale sous ciliaire, elle est préférée car elle peut être facilement effectuée chez les patients présentant un œdème périorbitaire remarquable ou un chémosis conjonctival. Elle est utilisée en association à une incision cutanée sous sourcilaire inférieure en cas de décompression de la paroi latérale. Nous avons utilisé une décompression osseuse pour 11 yeux associée à une résection de la graisse extraconique pour 4 yeux. La décompression osseuse inclut le plancher de l'orbite (2 yeux), la paroi latérale et médiale (3 yeux), la paroi inféromédiale (5 yeux) et les 3 parois de l'orbite (2 yeux). La décompression médiale est une technique non dénuée de complication, elle entraine une restriction de l'oculomotricité [16]. Dans notre série, on a constaté une complication post opératoire chez un patient à type de diplopie de novo en réalisant la décompression de la paroi médiale, une réduction de l'exophtalmie et amélioration des symptômes chez 3 patients. Une persistance de lagophtalmie post opératoire a nécessité le recours à un allongement palpébral ultérieur chez 4 yeux.

**Allongement palpébral par injection de toxine botulique**: Plusieurs études [17, 18] ont rapporté l'effet favorable de l'injection transcutanée de toxines botuliques. Les doses thérapeutiques sont variables [19] dépendants de la sévérité de la rétraction palpébrale. Nous avons 2 patients (4 yeux) qui ont eu des injections de Botox par voie conjonctivale au stade de fibrose de la maladie. La toxine botulique est une alternative efficace mais son effet est transitoire, des injections répétées sont nécessaires, une sous ou sur correction est possible, une altération de l'élévation ou paralysie du muscles orbiculaire peut survenir [11]. Nous n'avons pas noté de complication dans notre étude. Une amélioration transitoire dans 2 yeux pour une forme de rétraction palpébrale modérée a été constatée mais l'injection s'est révélée sans effet pour 2 yeux présentant une rétraction persistante et lagophtalmie après chirurgie de décompression. Ces résultats ont été expliqué probablement par une injection superficielle de la toxine avec un mauvais repérage du site d'injection entre bord sup du tarse et jonction aponévrose du RPS et müller et par la variabilité de réponse inter individuelle.

**Chirurgie palpebrale**: La correction de la rétraction palpébrale par voie chirurgicale nécessite une récession des rétracteurs palpébraux. La méthode de Mourits [20] consiste en une désinsertion du muscle releveur de la paupière supérieure ainsi que le muscle de Muller puis suture de l'aponévrose et du tarse par des points avec ajustement du contour palpébral [15]. On a utilisé dans notre population la voie antérieure pour la paupière supérieure dans 3 yeux avec dissection du muscle releveur de la paupière supérieure et son allongement par l'aponévrose temporale et on a essayé d'ajuster le niveau de la paupière supérieure dans un œil seulement en réalisant une blépharotomie. L'abord de paupière inférieure se fait par désinsertion de la conjonctive et des rétracteurs du bord tarsal par un abord postérieur mais contrairement à la paupière supérieure l'interposition d'intercalaire est nécessaire pour offrir une rigidité pour supporter la paupière inférieure contre la gravité. En effet, on a corrigé la rétraction de paupière inférieure dans deux yeux par simple blépharoplastie dans un œil avec greffe dermique du quadrant supéroexterne de la fesse qui a été repris en en raison de nécrose post opératoire en utilisant une greffe de lambeau nasogénien au tissu sous cutané et par allongement de paupière inférieure d'un œil par l'aponévrose temporale.

**Orbitopathie dysthyroidienne sévères neuropathie optique**: Des études très limitées se sont intéressées à l'efficacité du traitement IV des stéroïdes dans la neuropathie optique [4, 6, 21–24]. Il n'existe pas de protocole standard dans la littérature; il existe un manque de recommandation et chaque auteur défend son attitude. Selon l'EUGOGO, le traitement de 1ére ligne consiste généralement en une forte dose de glucocorticoïde (500-1000mg) de méthylprednisolone pour 3 jours consécutifs ou des jours alternés durant une semaine. Cette cure peut être répétée après une semaine et elle est efficace chez 40% des patients avec récupération d'une vision normale [4]. Si pas de réponse après cette cure, une décompression osseuse doit être envisagée. Dans notre série, on a utilisé 2 protocoles différents pour le traitement de la NO soit 1g/jour pendant 3 jours consécutifs avec relais aux corticoïdes oraux soit 4 cures de 500 mg pendant 3 jours consécutifs espacés de 4 semaine d'intervalle. Des études randomisées contrôlées [25, 26] montraient l'efficacité de combiner la cyclosporine avec les glucocorticoïdes par voie orale par rapport à chacun des traitements utilisé seul chez les patients avec OD sévère. Le recours à la cyclosporine dans notre série a été réalisé chez deux patients et a montré résorption de l’œdème papillaire chez un patient et sans effet chez l'autre avec recours à la chirurgie. Pour tous les patients avec neuropathie optique inclus dans notre étude, une décompression orbitaire a été indiquée chez 4 patients pour persistance d'un œdème papillaire après corticothérapie (1oeil) ou début d'installation de pâleur papillaire avec persistance des altérations périmétriques (5 yeux). L'acuité visuelle finale dans notre étude était ≥ 6/10 dans 41.2% des yeux, 4 yeux ont une disparition complète des anomalies périmétriques. Le fond dœil révèlait la persistance d'un œdème papillaire dans un oeil et une pâleur papillaire dans 2 yeux. Le reste des papilles étaient normales (82.4%).

**Atteinte cornéenne:** L’évolution de nos cas d’abcès a été marquée par une cicatrisation de l'ulcère laissant une opacité séquellaire chez 2 patients, un nettoyage de l'abcès avec persistance d'ulcère trophique chez un patient qui a été expliqué par la survenue d'ulcère sur cornée décompensée et l'hypotropie résiduelle qui favorise l'exposition cornéenne. Un cas a été rapporté dans la littérature [27] d'ulcère cornéen trophique non amélioré par antibiotique topique, une greffe de membrane amniotique a été réalisée avec cicatrisation complète de l'ulcère au prix de zone de fibrose. La membrane amniotique est une bonne alternative dans ces situations, elle constitue un lit pour migration épithéliale, stimule la différenciation cellulaire et diminue le processus inflammatoire.

Malgré les limites de notre étude, elle constitue un premier état des lieux qui peut aider à tracer les grandes axes d'amélioration de la prise en charge de l'orbitopathie dysthyroidienne. Une étude prospective incluant un nombre plus large de participants doit être prévue.

## Conclusion

La prise en charge ophtalmologique de l'orbitopathie dysthyroidienne ne repose pas sur un consensus. Il existe plusieurs protocoles thérapeutiques et chacun défend son attitude. L'orbitopathie dysthyroidienne soulève des problèmes concernant le diagnostic de neuropathie optique et la prise en charge thérapeutique avec absence d'uniformité. Ces problèmes ne peuvent être redressés qu'après une validation par des études cohortes à grandes échelles.

### Etat des connaissances actuelle sur le sujet

- L'étiologie de l'orbitopathie dysthyroidienne est non connue;
- Les formes sévères menacent le pronostic visuel;
- Le traitement corticoïde a fait ses preuves dans la phase aigue de la maladie.

### Contribution de notre étude à la connaissance

- Le facteur le plus significativement associé à la neuropathie optique est la compression au niveau de l'apex orbitaire;
- La prise en charge de l'orbitopathie dysthyroidienne n'est pas codifiée;
- Les résultats thérapeutiques par corticoïdes ou décompression osseuses sont variables.

## Conflits d'intérêts

Les auteurs ne déclarent aucun conflit d'intérêts.

## References

[1] Sadoul JL (2011). L'ophtalmopathie thyroïdienne à l'heure de l'European Group On Graves Orbitopathy (EUGOGO). Presse Médicale..

[2] Asman P (2003). Ophthalmological evaluation in thyroid-associated ophthalmopathy. Acta Ophthalmol Scand..

[3] Dolman PJ (2012). Evaluating Graves' orbitopathy. Best Pract Res Clin Endocrinol Metab..

[4] Currò N, Covelli D, Vannucchi G (2014). Therapeutic Outcomes of High-Dose Intravenous Steroids in the Treatment of Dysthyroid Optic Neuropathy. Thyroid..

[5] McKeag D, Lane C, Lazarus JH (2007). Clinical features of dysthyroid optic neuropathy: a European Group on Graves' Orbitopathy (EUGOGO) survey. Br J Ophthalmol..

[6] Wakelkamp IMMJ, Baldeschi L, Saeed P (2005). Surgical or medical decompression as a first-line treatment of optic neuropathy in Graves' ophthalmopathy: a randomized controlled trial. Clin Endocrinol (Oxf)..

[7] Al-Bakri M, Rasmussen AK, Thomsen C (2014). Orbital Volumetry in Graves' Orbitopathy: muscle and Fat Involvement in relation to Dysthyroid Optic Neuropathy. ISRN Ophthalmol..

[8] Barrett L, Glatt HJ, Burde RM (1988). Optic nerve dysfunction in thyroid eye disease: CT. Radiology..

[9] Anderson RL, Tweeten JP, Patrinely JR (1989). Dysthyroid optic neuropathy without extraocular muscle involvement. Ophthalmic Surg..

[10] Chan LL, Tan H-E, Fook-Chong S (2009). Graves ophthalmopathy: the bony orbit in optic neuropathy, its apical angular capacity and impact on prediction of risk. AJNR Am J Neuroradiol..

[11] Bartalena L, Baldeschi L, Boboridis K (2016). The 2016 European Thyroid Association/European Group on Graves' Orbitopathy Guidelines for the Management of Graves' Orbitopathy. Eur Thyroid J..

[12] Kahaly GJ, Pitz S, Hommel G (2005). Randomized, single blind trial of intravenous versus oral steroid monotherapy in Graves' orbitopathy. J Clin Endocrinol Metab..

[13] Marcocci C, Marinò M (2012). Treatment of mild, moderate-to-severe and very severe Graves' orbitopathy. Best Pract Res Clin Endocrinol Metab..

[14] Zang S, Ponto KA, Kahaly GJ (2011). Clinical review: intravenous glucocorticoids for Graves' orbitopathy: efficacy and morbidity. J Clin Endocrinol Metab..

[15] Eckstein A, Schittkowski M, Esser J (2012). Surgical treatment of Graves' ophthalmopathy. Best Pract Res Clin Endocrinol Metab..

[16] Ben Simon GJ, Wang L, McCann JD (2004). Primary-gaze diplopia in patients with thyroid-related orbitopathy undergoing deep lateral orbital decompression with intraconal fat debulking: a retrospective analysis of treatment outcome. Thyroid Off J Am Thyroid Assoc..

[17] Uddin JM, Davies PD (2002). Treatment of upper eyelid retraction associated with thyroid eye disease with subconjunctival botulinum toxin injection. Ophthalmology..

[18] Scott AB (1984). Injection treatment of endocrine orbital myopathy. Doc Ophthalmol Adv Ophthalmol..

[19] Costa PG, Saraiva FP, Pereira IC (2009). Comparative study of Botox injection treatment for upper eyelid retraction with 6-month follow-up in patients with thyroid eye disease in the congestive or fibrotic stage. Eye Lond Engl..

[20] Mourits MP, Sasim IV (1999). A single technique to correct various degrees of upper lid retraction in patients with Graves' orbitopathy. Br J Ophthalmol..

[21] Lucarelli MJ, Shore JW (1996). Management of thyroid optic neuropathy. Int Ophthalmol Clin..

[22] Guy JR, Fagien S, Donovan JP (1989). Methylprednisolone Pulse Therapy in Severe Dysthyroid Optic Neuropathy. Ophthalmology..

[23] Panzo GJ, Tomsak RL (1983). A Retrospective Review of 26 Cases of Dysthyroid Optic Neuropathy. Am J Ophthalmol..

[24] Hart RH, Kendall-Taylor P, Crombie A (2005). Early response to intravenous glucocorticoids for severe thyroid-associated ophthalmopathy predicts treatment outcome. J Ocul Pharmacol Ther Off J Assoc Ocul Pharmacol Ther..

[25] Kahaly G, Schrezenmeir J, Krause U (1986). Ciclosporin and prednisone v, prednisone in treatment of Graves' ophthalmopathy: a controlled, randomized and prospective study. Eur J Clin Invest..

[26] Prummel MF, Mourits MP, Berghout A (1989). Prednisone and cyclosporine in the treatment of severe Graves'ophthalmopathy. N Engl J Med..

[27] Heinz C, Eckstein A, Steuhl K-P (2004). Amniotic membrane transplantation for reconstruction of corneal ulcer in graves ophthalmopathy. Cornea..

